# Abstract of Lettsomian Lectures on the Treatment of Some of the Forms of Valvular Disease of the Heart

**Published:** 1883-03

**Authors:** Arthur Ernest Sansom

**Affiliations:** M. D. Lond., F. R. C. P., Physician to the London Hospital, Senior Physician to the North-Eastern Hospital for Children


### Selections.

ABSTRACT OF LETTSOMIAN LECTURES ON THE TREATMENT OF SOME OF THE FORMS OF VALVULAR DISEASE OF THE HEART. Delivered before the Medical Society of London. By ARTHUR ERNEST SANSOM, M.D. Lond., F.R.C.P., Physician to the London Hospital, Senior Physician to the North-Eastern Hospital for Children.

LECTURE II.—MITRAL REGURGITATION. *Morbid Anatomy—Clinical Study—Regurgitation in Adynamia, in Acute Fevers, in Rheumatism and in Degeneration—Treatment General and Special—Action of Digitalis, Belladonna, Caffeine, Convallaria majalis, etc.*

I have to ask your attention this evening to the subject of the treatment of various conditions of disease associated with a certain imperfection in the mechanism of the heart—an imperfection of closure of the left auriculo-ventricular orifice at the time of systole, occasioning the reflux of a portion of the contents of the left ventricle into the left auricle, the mitral valve being inadequate to close the orifice. Pathological anatomy teaches that such result may be brought about by several varieties of morbid change :

1. By dilatation of the left ventricle without structural disease of the valve ; so the free borders of the curtains are drawn upon by their circumferential attachments, and prevented from a perfect apposition in systole.

2. By diseased conditions of the valve-curtains, the tendinous cords and fleshy columns, induced by endocarditis, and the changes consecutive thereto.

3. By rupture of the valve-curtains, cords, or columns, and their consequent incompetence. It has been supposed that rupture may occur from sudden strain in a healthy heart, but Drs. Wilks and Moxon have given strong reasons for the conclusion that there must have been some dilatation, at least, of the left ventricle previously. They consider that this accident is not of infrequent occurrence.

4. By atheromatous disease, patches of which may be observed upon the valve with consecutive degenerative change, rendering it inadequate.

5. By ulceration of portions of the valve and the surrounding structures.

Mitral regurgitation is not, however, wholly to be interpreted by pathological anatomy; it is to clinical investigation that we must chiefly look for guidance. Of this condition, a murmur at the left apex of the heart with the systole is the sign almost, though not quite, pathognomonic. The only condition with which it is likely to be confounded is, in my opinion, pericardial roughening at or about the apex. I have never known a difficulty about the differential diagnosis in the case of adults, but I have observed such difficulty several times in children. In cases of children I have repeatedly said that the quality, character, and situation of a systolic apicial murmur will not declare with precision whether there be exocardial or endocardial disease. Combined clinical and necroscopic observation soon convinces us that, in certain cases, wherein we have determined from such physical sign that mitral regurgitation existed during life, no lesion indicating inadequacy of the mitral valve to close its orifice has been discovered after death. Moreover, in some cases, where we have not only observed the sign mentioned, but where the whole category of signs, symptoms, and consecutive changes which experience has taught us to associate with mitral inadequacy has been present, the necropsy has demonstrated no determinate lesion at the orifice.

It will best serve a practical purpose, I think, if we divide the cases in which the signs indicating mitral regurgitation are evident into clinical groups, discussing the bearing of the collateral phenomena upon treatment in each group. We shall thus consider the cases just as we meet with them in practice.

I. A case presents itself, manifesting signs indicating mitral regurgitation in the subject of marked anæmia. We have to inquire whether or no there has been antecedent disease, leading up to organic change at the mitral orifice. Supposing such signs are not in evidence, have we a right to assume that actual mitral regurgitation can be induced by the condition of anæmia without concurring causes? The answer is, in my opinion, undoubtedly in the affirmative. In cases of anæmia and chlorosis, a murmur is sometimes heard exactly in the site of that indicating mitral regurgitation. Assuming that, in these cases, there is a veritable regurgitation, how is such brought about? The explanation, is, I think, given by the careful experiments conducted by Ludwig and Hesse at Leipzig, which have been admirably summarized by Dr. Donald Macalister (Remarks on the Form and Mechanism of the Heart). The mechanism for the closure of the left auriculo-ventricular orifice does not reside in the valve-curtains alone; the surrounding muscles of the ventricle have an active share, not merely in floating up the valve-curtains, but in reducing the size of the aperture which these valve-curtains have to close. It is not that the orifice is dilated, but that it is insufficiently contracted, the aid of the muscles of the wall of the ventricle which normally produce such contraction being lost.

It is important, in regard to treatment, to differentiate mitral regurgitation due to disease of the valves, from that due to adynamia of ventricle, supposing a systolic-apicial movement to be manifest in a markedly anæmic snbject. The two signs I would most rely on as pointing to an anæmic causation of the murmur are (1) an absence of notable cardiac dilatation; (2) a heightened tension in the systemic arteries. I have never known in these cases any marked improvement follow the administration of the usual cardiac tonics, such as digitalis and iron. In the cases attended with hæmorrhage, it is, of course, of the first importance to arrest this at its source. Rest, and the administration of assimilable food, are no less important indications. In this connection, I may call attention to the great value I have observed to attach to supplementary alimentation by the rectum in such cases. I have long tried the plan of using defibrinated ox-blood for a nutrient enema, as advocated by my friend Dr. A.

H. Smith, of New York. In comparing results, however, with those in which artificially digested food has been employed, I felt that the balance of evidence is in favor of the latter plan. I have had prepared mixed peptone enemata—beef, milk, and farinaceous food—which have been proved to preserve a perfectly good condition for long periods. These have the advantage of being available at a moment's notice, it being only needful to render them diffluent with warm water. From two to four ounces are injected slowly into the rectum, and repeated every three or four hours. In many cases, I have caused to be added the dry ox-blood (sanguis bovinus exsiccatus), in the proportion of a drachm to the ounce. I have lately, however, adopted a simpler plan with good results; using, instead of peptoned food, equal parts of warm milk and cod-liver oil, as a nutritive enema. In the treatment of cases of idiopathic anæmia, I have found no drug-treatment so efficient as the administration of arsenic (Fowler's solution in small doses gradually increased). I have observed, as has been recorded by others, complete recovery, with the disappearance of the cardiac murmur, under such treatment, combined with rest and careful nutrition.

II. We will now assume that a systolic apex-murmur is present in a patient showing signs of *a neurosis of the cervical sympathetic*. It has been frequently noted that a murmur at the apex has existed in the subjects of exophthalmic goitre (Graves's or Basedow's disease); yet, on post mortem examination, no disease at the mitral orifice has been discovered. In these cases, anæmia may be present, but not of necessity. It is not causally related with the phenomena. Organic heart-disease may coexist, but such coincidence is rare. It is important to recognize, especially with regard to treatment, that, in the subjects of Graves's disease, mitral regurgitation occurs without valvular lesion. The record of fatal cases in which disease of the cervical sympathetic ganglia has been actually demonstrated in Graves's disease, is now tolerably extensive. Trousseau, Cruise, and McDonnell, Reith, and Shingleton Smith, have recorded cases in which some of the ganglia (usually the inferior cervical) have been enlarged, atrophied, or degenerated.

As regards the cases which I have seen, ordinary tonics and digitalis have been of very little benefit; but great improvement has followed galvanization of the cervical sympathetic. I have employed the continuous current, from twenty to forty elements (Leclanché). One pole may be placed behind the lower jaw in front of the sterno-mastoid, and the other either at a corresponding point of the opposite side or at the nape of the neck, right or left of the vertebra prominens, or above the sternum at the inner edge of the insertion of the sterno-mastoid.

III. I now turn to a third group of cases, and assume that the indications of mitral regurgitation are manifest during the *evolution of certain fevers.* In the course of typhoid fever, for example, a systolic murmur may be discovered at the apex. There is no history of its existence before the attack, but it has arisen during the course of the disease. The murmur is an evanescent one. To what is it due? The changes are, according to M. Hayem's observations, not in the endocardium nor pericardium, but in the muscle of the heart. In fatal cases, the muscular fibers present a granular and fatty degeneration, or a special form of vitreous degeneration; the areas of morbid change are disseminated in an irregular manner here and there throughout the cardiac muscle. There are, besides, a multiplication of the muscular nuclei and aggregation of cellular elements; in fact the disease is a form of myocarditis. It is, I think, sufficiently proven, that the murmur occasionally heard at the apex in cases of typhoid is due to regurgitation, on account of imperfect apposition of the valves of left or right sides from enfeeblement, by disease, of the muscular fibers in certain areas of the heart wall. It does not appear that the occurrence of such murmur renders the prognosis more grave, but sudden death from myocarditis in all probability may occur in typhoid, without any special evidence of direct cardiac impairment previously. Its occurrence, however, should make us watchful, and cases presenting any of the phenomena indicating myocarditis in typhoid should be observed and treated with a view of preventing subsequent dilatation. Analogous myocarditis has been described in variola (by MM. Desnos and Huchard), and in severe forms of intermittent fever as observed in Africa (by M. Vallin).

It is obvious that a recognition of the nature of the alteration which produces a mitral regurgitant murmur in these cases must have an important bearing on treatment. We need not fear that endocarditis has arisen as a complication, nor have we to debate as to an anti-rheumatic plan of treatment. The indication is to keep the disturbed muscle of the heart as tranquil as possible, and, of course, to promote as good a nutrition as the circumstances will permit.

IV. I now come to the fourth group, and assume that a murmur indicating mitral regurgitation is observed in the subjects of *acute or subacute rheumatism.* Attention has been frequently drawn to the fact that murmurs may arise in the course of evolution of the disease, and yet disappear, and patients may be supposed to be free from cardiac complication. I have, in my former lecture, deprecated this as a too hasty conclusion. It may be well to inquire, in the first place, what is the probable nature of these transitory or evanescent murmurs, which are by no means uncommon, for they occur, as the statistics of the London Hospital for 1880 and 1881 show, in about 10 per cent. of the cases. Rheumatism is a disease notably attended with anæmia. The evidence collated for me by Dr. Gabbett as to the site of such transient murmurs is, I think, against this view. It is well known that the murmurs heard in connection with anæmia, though sometimes heard at the apex, and indicating mitral regurgitation, are far more frequently audible at the base over the site of the pulmonary artery or aorta; even when heard at the apex, they are usually accompanied by other murmurs at the base. In rheumatism, however, the usual site of the evanescent murmur is the apex. The totals for 1881 show as follows: Transient murmurs in mitral area, 15; at base and apex, 7; in aortic area, 5; in pulmonic area, 3.

It would appear that a murmur which might suggest an anæmic causation is almost confined to a first attack of rheumatism. After two or more attacks, no basic transitory murmurs are recorded. Then, as regards the transient systolic murmur in the mitral area, we may ask whether it may be due to myocarditis. If so, it does not resemble in associated phenomena the murmur observed in typhoid, etc. The peculiar perturbations of rhythm are not recorded, and it would appear probable that, if there be myocarditis, it does not occur in disseminated areas as in typhoid. May it not be that the temporary regurgitation is due to a localized myocarditis developed in the neighborhood of a swollen valve or inflamed endocardium? Thus, though the swollen valve might not be in itself incompetent, a temporary incompetence would be produced by the impairment of the force of the muscle. As the myocarditis subsided, the valve would again become competent, but probably, in many instances, to present a renewed imperfection when the swelling, in the course of time, has given rise to fibrous change and consequent shrinking. I draw attention to this as a caution as to the expression of any opinion that a valve is sound after a murmur developed during the early stages, and disappearing during the latter period of rheumatic fever.

Supposing that mitral regurgitation is left after rheumatic endocarditis, it is well known that compensation may be effected, and the health of the patient be preserved for very long periods with no subjective symptom of cardiac unsoundness. The chief factor in inducing such compensation is a (conservative) hypertrophy of the right ventricle, and the sign of such compensation (supposing the amount of blood regurgitating to be not very small) is an accentuated second sound over the pulmonary semilunar valves. If we are satisfied that there be due compensation, medicinal treatment may be entirely unnecessary. I have no doubt that a vast amount of injury has been done to patients by a shaking of the head of the auscultator over the subject of a mitral murmur, who perhaps was no worse at the time of examination than he was ten, twenty, or thirty years before, and who might continue uninfluenced for harm by his cardiac complication all his days. He should be cautioned against strain, exposure, irregularities of diet, etc.; he may be better occasionally for treatment by iron tonics, cod-liver oil, or strychnine, but any special cardiac treatment is out of place.

Not so, however, if there be evidence that compensation is beginning to fail. I will pass in brief review the chief agents which are of service in such case.

I. Digitalis is *facile princeps* of drugs in the treatment of imperfect compensation. A little over a suitable dose, however, may induce nausea, vomiting, anuria, irregularity of pulse, and, instead of slowing, an enhanced rapidity of heart-action. Whilst a dose which produces favorable result is constant and discoverable, in regard to a large majority of patients, in a minority, such dose is inconstant and even unattainable.

As regards the preparation used, we may have differences of results, and we know that, as in the case of so many vegetable products, the energy of different samples may vary. Practically, I consider the tincture most reliable, and that usually in small doses, five minims to ten minims, increased only in exceptional cases, and then occasionally reduced; next in value I considered the powdered leaves (half a grain to two grains) the combination of which with alkalies I shall hereafter consider.

In some cases, even by increasing the dose, no apparent influence appears to be exerted by the drug; then digitalis, especially when hypodermically injected, I have observed to give, in many cases, good results. The digitalis hitherto prepared has probably scarcely ever been the pure alkaloid. The usual dose for hypodermic administration is one-fiftieth of a grain.

When the right ventricle has dilated so far that there is marked tricuspid regurgitation, the beneficial action of digitalis is by no means so decided. Nevertheless (especially when purgatives are also administered), the signs of tricuspid regurgitation may pass away. In other cases, no such favorable result attends. In fact, as *à priori* considerations might suggest, any increased force of systole which the digitalis may bring about serves the more to urge back the blood through the imperfect tricuspid orifice into the venous channels. But yet I have seen good results when the administration of digitalis has been combined with abstraction of blood by leeches or cupping to relieve venous engorgement.

II. Belladonna is, I think, only useful in the treatment of failure of compensation in cases of mitral regurgitation, when combined with, or occasionally substituted for, digitalis. Belladonna, like digitalis, increases the power of systole and raises the arterial tension. As Dr. Lauder Brunton has shown, it paralyzes the cardiac terminals of the vagus, and reduces irritability by an anæsthetic effect on the sensory nerves of the heart. Very useful occasionally, it by no means compares with digitalis for prolongd employment. The hypodermic injection of one-fiftieth of a grain of digitalis with one-eightieth of a grain of atropine I have found very satisfactory.

III. Casca.—A tincture made from the bark of the ordeal bark of West Africa has been employed as a substitute for digitalis. Dr. Lauder Brunton, in his Gulstonian Lectures for 1877, published the results of elaborate experiments as to its physiological action. In kind, this action appears much to resemble that of digitalis. Dr. Brunton has said: "Digitalis has hitherto been our great resort in mitral disease, but I think it probable that in casca we possess a drug more powerful still; at least, its effect upon the arterioles appears to be greater than that of digitalis, and it is quite possible that it may succeed in those cases of advanced mitral disease where digitalis fails." I have myself employed the tincture of casca, substitutively for digitalis, in a considerable number of cases, but I have never yet been able to convince myself that it has any more beneficial action in mitral disease.

IV. Caffeine.—Gubler, Shapter, Leech, Milliken, Brakenridge, Huchard, and others have recorded observations showing the action of caffeine (or its citrate) in cases of cardiac disease, especially where dropsy is a marked symptom. Some of the cases show very forcibly that a beneficial influence has been exerted by the drug. There are many apparently contradictory data as to the physiological action, but the cardinal points are, that it at first quickens, but soon after slows the heart's action, and that it acts in a very pronounced manner as a diuretic in cardiac dropsy. Dr. Brakenridge advises that digitalis be administered previously to or in conjunction with the citrate of caffeine, and that small doses (three grains) should be employed. M. Huchard, however, recommends that caffeine and not its citrate should be used, and that in larger doses (four to six grains). It produces diuresis more rapidly than digitalis, and has none of its nauseating effects. I have employed citrate of caffeine in substitution for digitalis without any marked benefit being manifest; indeed, I have found that in some cases it has induced insomnia. Nevertheless, I consider that the evidence is such that I should certainly employ it in many cases where, as in cardiac dropsy, a rapid diuretic effect is desirable.

V. Convallaria majalis.—This is the well-known lily-of-the-valley, long employed by the Russian peasantry as a remedy for dropsy. Professor Sée has shown that it has an action much resembling that of digitalis. An extract of the whole plant is employed in doses of from five to eight grains, three times a day. In cases of mitral regurgitation with severe symptoms, it entirely relieved the cardiac distress, and manifesting a decided diuretic action, removed the dropsy. Professor Sée considers that it may be used in all forms of heart failure, for it has none of the nauseating effects of digitalis, nor does it exhaust the contractility of the heart and arteries. I have employed it as a substitute for digitalis, and am convinced of its action in promoting a stronger ventricular contraction ; but I am not yet convinced of its superiority to digitalis.

VI. Morphia.—The hypodermic injection of morphia, as advocated by Dr. Clifford Allbutt, is a most valuable adjunct to the treatment of failure of compensation in cases of mitral regurgitation. I have found preparations of opium by the mouth generally disagree, but not so when the alkaloid is hypodermically injected. It is often very advantageous to combine the morphia with atropia or digitaline.

V. By no means all the cases which come before us showing mitral regurgitation are to be explained by the modes of causation we have hitherto discussed. In a considerable minority, such regurgitation is secondary to combined high tension in the aorta and arteries. It is important for prognosis and treatment to discriminate the cases of *mitral regurgitation due to heightened arterial tension.* In such, the apicial murmur is usually post-systolic, the signs of hypertrophy preponderate, the patient is usually, though not always, of middle or advanced age, the advent of signs has been gradual; often the arteries may be observed to be tortuous and hard. The most important signs however, are the discovery either of aortic disease, or of accentuation of the aortic second sound, with pulse of high tension. Chronic renal disease may be also manifested. When not so complicated, great improvement often follows a prolonged treatment by alkalies with iodide of potassium. A carefully regulated diet is most important, and those cases do best, I am convinced, who entirely abstain from alcohol. It is by no means infrequent to find a murmur of regurgitation brought about by such causes wholly disappear. Their epiphenomena are often to be successfully treated by the administration of nitro glycerine, or the inhalation of nitrate of amyl.—*British Medical Journal.*

